# A model for biased fractionation after whole genome duplication

**DOI:** 10.1186/1471-2164-13-S1-S8

**Published:** 2012-01-17

**Authors:** David Sankoff, Chunfang Zheng, Baoyong Wang

**Affiliations:** 1 Department of Mathematics and Statistics, University of Ottawa, Ottawa K1N 6N5, Canada

## Abstract

**Background:**

Paralog reduction, the loss of duplicate genes after whole genome duplication (WGD) is a pervasive process. Whether this loss proceeds gene by gene or through deletion of multi-gene DNA segments is controversial, as is the question of fractionation bias, namely whether one homeologous chromosome is more vulnerable to gene deletion than the other.

**Results:**

As a null hypothesis, we first assume deletion events, on either homeolog, excise a geometrically distributed number of genes with unknown mean *μ*, and a number *r *of these events overlap to produce deleted runs of length *l*. There is a fractionation bias 0 ≤ *ϕ *≤ 1 for deletions to fall on one homeolog rather than the other. The parameter *r *is a random variable with distribution *π*(·). We simulate the distribution of run lengths *l*, as well as the underlying *π*(·), as a function of *μ*, *ϕ *and *θ*, the proportion of remaining genes in duplicate form. We show how sampling *l *allows us to estimate *μ *and *ϕ*. The main part of this work is the derivation of a deterministic recurrence to calculate each *π*(*r*) as a function of *μ*, *ϕ *and *θ*.

**Conclusions:**

The recurrence for *π *provides a deeper mathematical understanding of fractionation process than simulations. The parameters *μ *and *ϕ *can be estimated based on run lengths of single-copy regions.

## Background

Whole genome doubling (WGD) creates two identical copies (*homeologs*) of each chromosome in a genome, with identical gene content and gene order. From this ensues the wholesale shedding of duplicate genes over evolutionary time through random *excision *- elimination of excess DNA - namely the deletion of chromosomal segments containing one or more genes, or through gene-by gene events such as epigenetic silencing and pseudogenization [1-6].

When a duplicate gene is lost, it may be lost from one copy (*homeolog*) of a chromosome or the other, but generally not both, because of the necessity of conserving function. This *fractionation *creates an interleaving pattern; the full original gene complement becomes apparent only by *consolidating *[5] the two homeologous single-copy regions. In most cases, there is a degree of bias, more genes being lost from one of the homeologous regions than the other [4-7]. Fractionation is an important process in many evolutionary domains, in particular the flowering plants, since it results in a genome that is highly scrambled with respect to its pre-WGD ancestor. For this reason as well, fractionation raises a number of interesting and difficult problems for comparative genomics.

The study of fractionation is basically a study of runs, that is runs of duplicate genes on two homeologous chromosomes alternating with runs of single-copy genes on one or both of these chromsomes. Because of the way these runs are generated biologically, and because they involve two chromosomes evolving in a non-independent way, standard statistical or combinatorial run analyses are not directly applicable.

In this paper, we present a detailed version of the excision model of fractionation with geometrically distributed deletion lengths, for which we previously analyzed a tractable, but biologically unrealistic, special case [8]. The key problem in this field is to determine *μ*, the mean of the hypothesized geometric distribution $\rho \left(\frac{1}{\mu },.\right)$, since this bears directly on the main biological question of the relative importance of random excision versus gene-by-gene inactivation. The relevant data consist of runs of single-copy genes (whose duplicates have been lost from the homeologous region) as well as runs of remaining duplicate pairs in two homeologous regions. The inference of *μ *is complicated since each run of *l *single copies may have been produced by an unknown number *r *of deletion events, either *r *= *l *events (the gene-by-gene model) or 1 ≤ *r *<*l *- 1 (the random excision model), and these *r *samples of the distribution *ρ *turn out not to be independent. Thus a fundamental aspect of finding *μ*, and hence $\rho \left(\frac{1}{\mu },.\right)$, is to derive *π*(*r*), the proportion of runs of single-copy genes with *r *terms, for *r *= 1, 2, ⋯.

A further complication arises from the way deletion events accumulate into longer runs of single-copy genes. The deletion of a certain number of duplicate genes may overlap the site of a previous deletion event on the *same *chromosome, but it is blocked by the functional constraint (mentioned above) as soon as it starts to overlap the site of a previous deletion event on the *homeologous *chromosome.

Another biologically important question is to determine *ϕ*, the proportion of deletion events that operate on one of the homeologous chromosomes, while a proportion 1 - *ϕ *operates on the other. We explored this question at some length in [4], but a detailed mathematical treatment of the effects of this "fractionation bias" remains to be done.

It is not difficult to simulate the fractionation process, but this gives little insight into its mathematical structure. Given that it is unlikely for any closed form of π to exist, nor for any simple computing formula, our goal here is to develop a recurrence for the distribution of π(*r*) for *r *= 1, 2, ⋯ as a function of *μ*, *ϕ *and *θ *(the proportion of duplicate pairs remaining in the genome versus single-copy genes).

This work is an attempt at creating a rigorous "null" model of duplicate loss, based on parameters *μ*, *ϕ *and *θ*. This should provide a principled basis for developing statistical tests on real WGD descendants, to see if the geometric excision hypothesis is acceptable and to see if fractionation is unbiased or not. We will not explicitly investigate the alternative hypothesis of gene-by-gene deletion, nor do we take chromosomal rearrangement events into account; our task here is simply to set up the null statistical model with a view to enabling useful statistical tests of hypothesis for this problem.

### The models

#### The structure of the data

The data on paralog reduction are of the form (G, *H*), where *G *and *H *are binary sequences indexed by **ℤ**, satisfying the condition that *g*(*i*) + *h*(*i*) > 0. This condition models the prohibition against deleting both copies of a duplicated gene. We may also assume that whatever process generated the 0s and 1s is homogeneous on **ℤ**.

The sequence *G *+ *H *consists of alternating runs of 1s and 2s. We denote by *p*(*l*), *l *≥ 1 the probability distribution of length of runs of 1s. For any finite interval of **ℤ **we denote by *f*(*l*), *l *≥ 1 the empirical frequency distribution of length of runs of 1s.

The use of **ℤ **instead of a finite interval is consistent with our goal of getting to the mathematical essence of the process, without any complicating parameters such as interval length. In practice, we use long intervals of at least 100,000 so that any edge effects will be negligible. See [4,8] for *ad hoc *ways of handling biological scale intervals.

#### The deletion events

Let *ϕ*, where 0 ≤ *ϕ *≤ 1, be the fractionation bias. We assume a continuous time process, parameter *λ*(*t*) > 0, only to ensure no two events occur at the same time.

• We start (*t *= 0) with *h*(*i*) = *g*(*i*) = 1 for all *i*.

• At any *t *> 0, consider any *i *where *h*(*i*) = *g*(*i*) = 1. With probability *λ*(*t*)*dt*, a *deletion event *occurs *anchored *at position *i*: we choose a positive number *a *according to a geometric variable **y **with parameter 1/*μ*, i.e., $P\left[y=a\right]=\gamma \left(a\right)=\frac{1}{\mu }{\left(1-\frac{1}{\mu }\right)}^{a-1},\;a\geq 1$.

• Then with probability *ϕ *we choose to carry out the deletion on *G*; with probability 1 - *ϕ*, on *H*.

• If the deletion is on *G *we convert *g*(*i*) = 0, *g*(*i *+ 1) = 0, ⋯, *g*(*i *+ *a *- 1) = 0 unless a "collision" occurs.

• One type of collision, *skippable *collision, arises when one or more of *g*(*i *+ 1), ⋯, *g*(*i *+ *a *- 1) is already 0. In this case we skip over the existing 0 values and continue to convert the next available 1s into 0s, until a total of *a *1s have been converted, or a collision of the second type is encountered.

• The second type of collision, *blocking *collision, arises when one or more of *h*(*i *+ 1), ⋯, *h*(*i *+ *a *- 1) (or a further term if skipping has already occurred during this event) is already 0. In this case, further conversions of 1s to 0s are blocked, starting with the first *g*(*x*) for which *h*(*x*) = 0.

Skippable collisions are a natural way to model the excision process, since deletion of duplicates and the subsequent rejoining of the DNA directly before and directly after the excised fragment means that this fragment is no longer "visible" to the deletion process. Observationally, however, we know deletion has occurred because we have access to the sequence *H*, which retains copies of the deleted terms. Blocking collisions are a natural way of modeling the constraint against deleting single-copy genes.

When the deletion event has to skip over previous 0s, this hides the anchor *i *and length *a *of previous deletion events. Denote by **r **the random variable indicating the total number of deletion events responsible for a run. Then, given **r **= *r*, the run length **z **is distributed as the sum of *r *geometric variables, which would result in a negative binomial distribution were these geometric variables independent. They are not, however, since events with large *a *tend to group together in runs with large *r*, while an event with small *a *is more likely to constitute by itself a run with *r *= 1 [8].

If we observe *G *at some point in time, as in the last pair of rows of Table 1, all we can observe are the run lengths of 0s and 1s. We cannot observe the a, *i *or *r*, while *t *and *λ*(*t*) are unknown and, as we shall see, only mathematical conveniences that are supplanted by *θ *in our calculations. The parameters about which we wish to make statistical inferences are the deletion length distribution parameter *μ*, and the fractionation bias *ϕ *since it is these quantities that are at the heart of the biological controversies about paralog reduction. This inference can only be based on the two observable quantities: the run lengths *l *and the proportion *θ *of remaining (undeleted) 1s.

**Table 1:** Deletions with skipping and blocking

Event	*i*	*a*	-7	-6	-5	-4	-3	-2	-1	0	1	2	3	4	5	6	7	8	*r*
Start			1	1	1	1	1	1	1	1	1	1	1	1	1	1	1	1	
			1	1	1	1	1	1	1	1	1	1	1	1	1	1	1	1	
1	-1	3	1	1	1	1	1	1	0	0	0	1	1	1	1	1	1	1	1
			1	1	1	1	1	1	1	1	1	1	1	1	1	1	1	1	
2			1	1	1	1	1	1	0	0	0	1	1	1	1	1	1	1	1
	-4	1	1	1	1	0	1	1	1	1	1	1	1	1	1	1	1	1	1
3	5	1	1	1	1	1	1	1	0	0	0	1	1	1	0	1	1	1	1,1
			1	1	1	0	1	1	1	1	1	1	1	1	1	1	1	1	1
4	4	3	1	1	1	1	1	1	0	0	0	1	1	0	0	0	0	1	1,2
			1	1	1	0	1	1	1	1	1	1	1	1	1	1	1	1	1
5			1	1	1	1	1	1	0	0	0	1	1	0	0	0	0	1	2
	-5	4	1	1	0	0	0	0	1	1	1	1	1	1	1	1	1	1	3

Five deletion events affecting two homeologous chromosomes, leading to two runs of single-copy genes. The fourth step illustrates the "skip" process, at *i *= 5 where the pre-existing deletion is incorporated into a longer run with *r *= 2. The fifth step shows how further deletion (at *i *= -1) and the "skip" process (to *i *= 2) are blocked when a single-copy gene is encountered (*i *= -1) on the homeologous chromosome. This creates a single-copy run with length *l *= 7 and *r *= 3, part on one chromosome, part on the other. Note that *r *is not observable from the genome data.

## Results

### Simulations to determine *π*

We carried out simulations on an interval of **ℤ **of length 100,000. This enabled us to use a discrete time process instead of the continuous time process on **ℤ**. The "anchors" for the deletion events were chosen at random among the currently undeleted genes. The remaining steps were carried out as described in the previous section and Table 1. Because each simulation run samples thousands of deletions, it sufficed to do 100 runs for each value of the parameters *μ *and *ϕ *studied.

**Figure 1:**
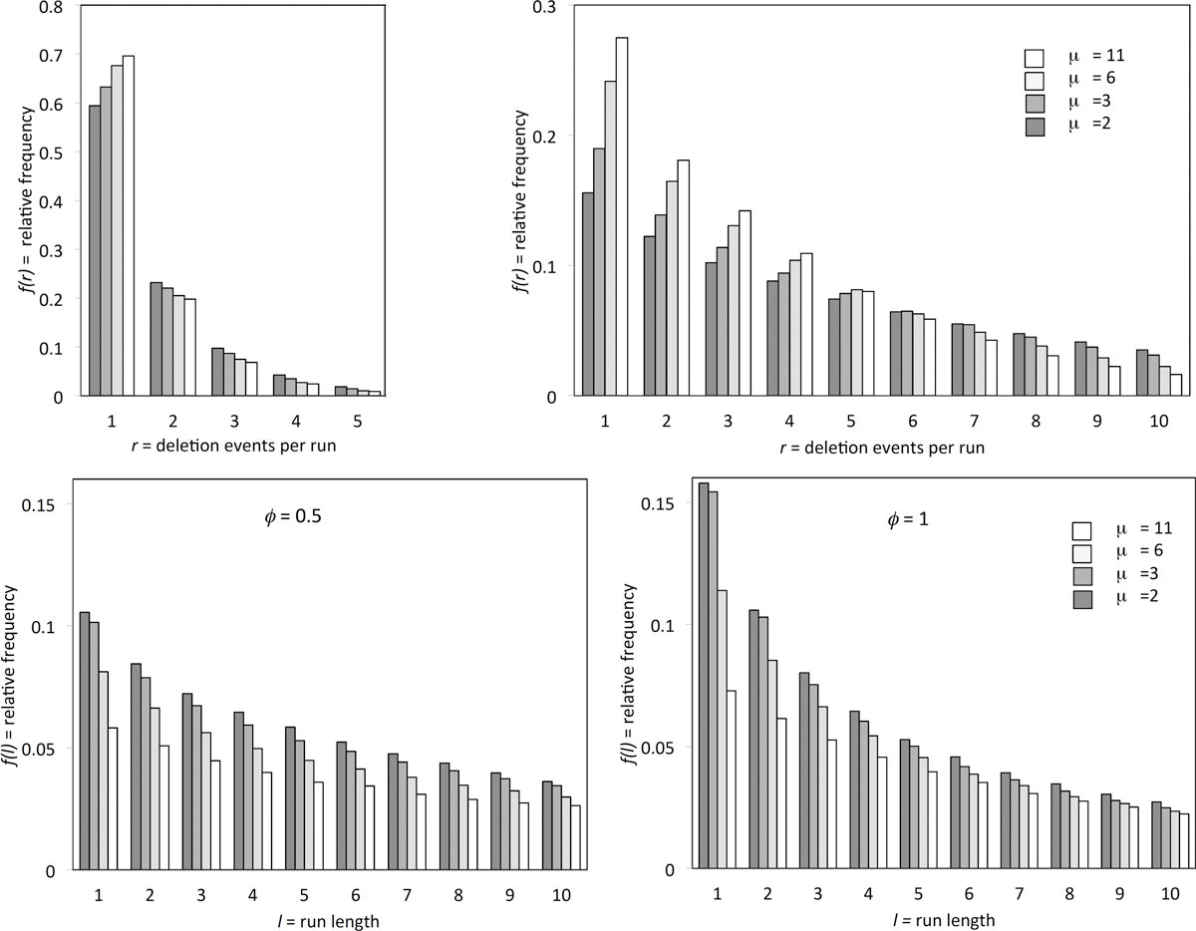
**Simulations of events per run and run length. **Distribution of number of deletion events *r *composing each run when 1 - *θ*, the proportion of sequence deleted, is 0.5 (top left) and 0.9 (top right). *ϕ *= 0.5 in both cases. Distribution of run length for for *ϕ *= 0.5 (bottom left) and *ϕ *= 1 (bottom right). For visibility, all diagrams show highest frequency parts of the distribution only.

The top row of Figure 1 compares *π*(*r*) when *θ *= 0.5 and *θ *= 1, for *μ *= 2, 3, 6, and 11, when *ϕ *= 0.5. We can see that the number of deletion events contributing to a run is somewhat dependent on *μ *when half of the the sequence has been deleted, but is strongly dependent when 90% has been deleted. In the bottom row, the graph on the left shows that run length l is distributed very differently for *μ *= 2, 3, 6 and *μ *= 11, when the proportion of the sequence deleted is exactly the same. This strongly suggests that observing the run length distribution and the overall proportion of deletions should allow us to infer *μ*. Moreover the shape of these distributions is sensitive to *ϕ*.

We mention that any edge effects in our simulation are negligible. Whether we work with *G *and *H *on an interval of **ℤ **of length 100,000 or, as previously [8], length 300,000, gives virtually the same results.

**Figure 2:**
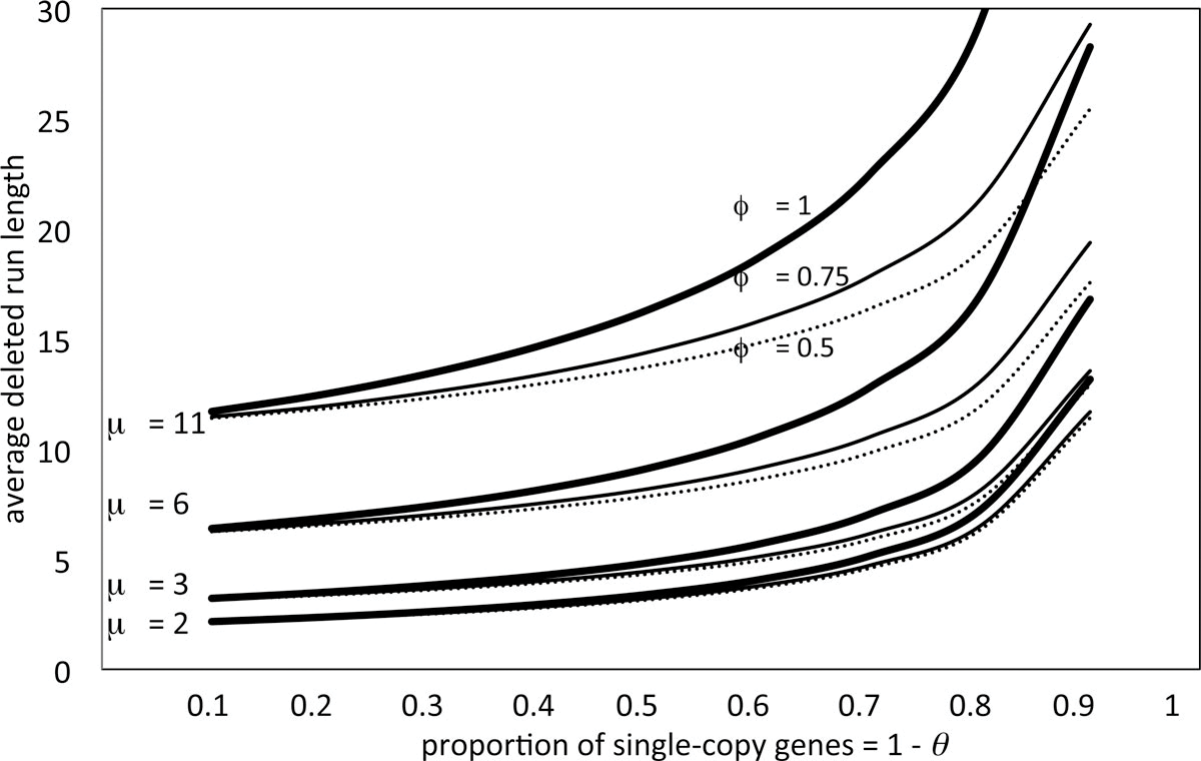
**Dependance of run length on deletion parameters. **Average length of run of single copy genes in for *ϕ *= 0.5, 0.75, 1.0, for *μ *= 2, 3, 6, 11.

Figure 2 shows the relationship, for three values of the fractionation bias *ϕ *and for a range of values of *μ*, between the proportion of genes deleted, on one chromosome or the other, and the average run length. This confirms that average run length and overall proportion of deletion *θ*, both observable, can be used to infer *μ *rather accurately, and to infer *ϕ*, perhaps with somewhat less precision. The latter parameter can, however, be inferred from the shape of the run length distribution in Figure 1 (bottom) or estimated directly from the proportion of single-copy genes on each homolog.

### A recurrence for *π*(*r*)

We are interested in inferring *μ *from the observed distribution of run lengths and the proportion *θ *of undeleted terms, i.e., undeleted genes. At the outset *θ *= 1. As *t *→ ∞, *θ *→ 0. We are not, however, interested in *t*, since it is not observable and any time-based inference we can make about *μ *will depend only on run lengths and *θ *in any case. On the other hand, *r*, the number of deletion events per run is an interesting variable since we can assume run length is close to *rμ *on average, at least for small values of *θ*, and we can model the evolution of *r *directly We consider the distribution *π *as a function of *μ*, *ϕ *and *θ*.

**Figure 3:**
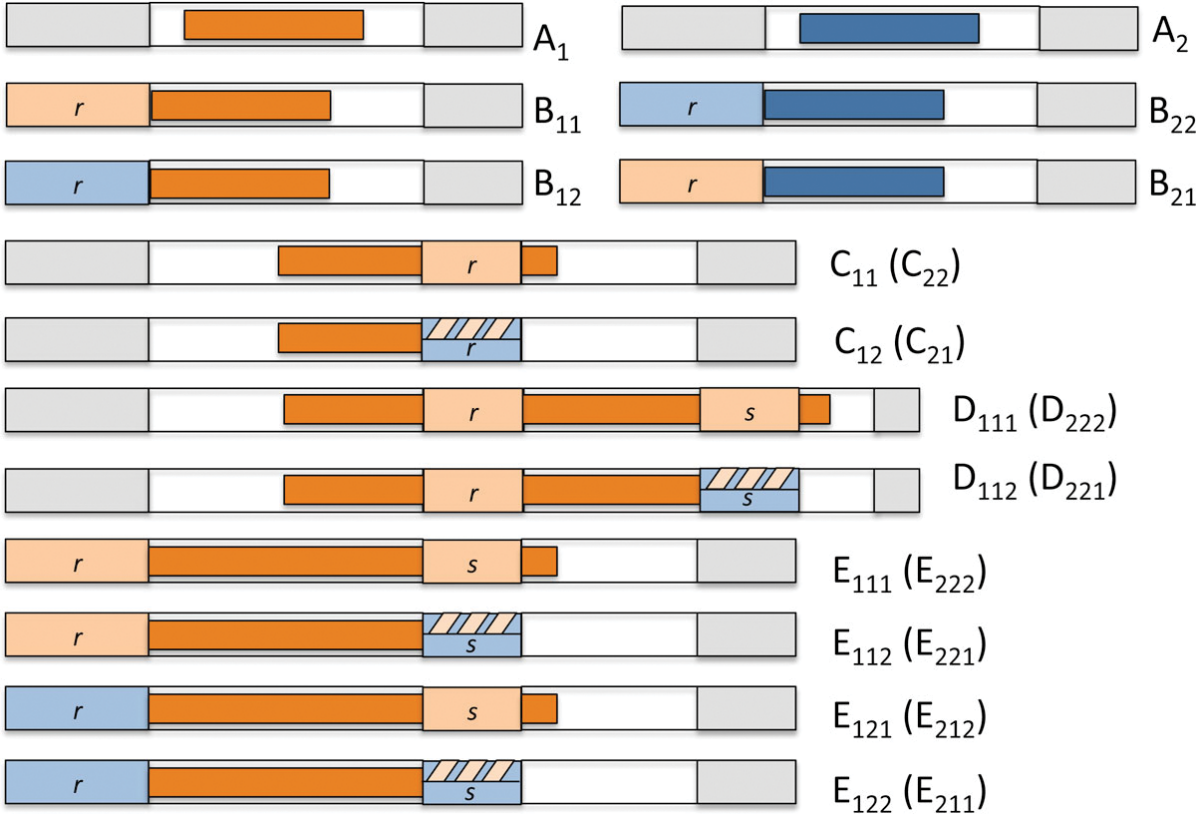
**Types of event.** Types of deletion event affecting less than three pre-existing runs. Red and blue shading distinguishes between deletions from the two homeologous chromosomes. Grey areas represent previous deletions from either chromosome. White area indicates run of undeleted terms. Lightly shaded area indicates run of previously deleted terms. Darker area represents current deletion event. Hatched striped area above lightly shaded area indicates either previous deletions from both homeologous chromosomes, or only from the homeolog not affected by the current deletion. A: creates one new run with *r *= 1. B: lengthens left hand run to *r *+ 1 events. C: lengthens right hand run to *r *+ 1 events. D and E: merge two runs to create a single run with *r *+ *s *+ 1 deletion events.

As *π *changes, probability weight is redistributed among several types of run:

1. new runs (*r *= 1) falling completely within an existing run of undeleted terms, not touching the preceding or following run of deleted terms, type A in Figure 3,

2. runs that touch, overlap or entirely engulf exactly one previous run of deleted terms with *r *≥ 1, thus lengthening that run to *r *+ 1 events, types B and C in Figure 3,

3. runs that touch, overlap or engulf, by the skipping process, two previous runs of *r*_1 _and *r*_2 _events respectively, creating a new run of *r*_1 _+ *r*_2 _+ 1 events, and diminishing the total number of runs by 1, including types D and E in Figure 3,

4. runs that touch, overlap or engulf, by the skipping process, *k *> 2 previous runs of of *r*_1_, ⋯, *r*_*k *_events respectively, creating a new run of *r*_1 _+ ⋯ + *r*_*k *_+ 1 events, and diminishing the total number of runs by *k *- 1, not illustrated in Figure 3. Case 3 above may be considered a special case of this for *k *= 2 and Case 2 for *k *= 1.

The first process, involving a deletion event of length *a *requires a run of undeleted terms of at least *a *+ 2. What can we say about runs of undeleted terms? We know that runs of deleted terms alternate with runs of undeleted terms, so that there is one run of the former for each of the latter. The mean lengths  and $\bar{v}$ of the deleted runs and the undeleted runs, respectively, should satisfy:

(1) $$\bar{v}=\frac{\theta }{1-\theta }ū.$$

The distribution *ρ*(*l*) of lengths of the undeleted runs is assumed to be geometric. Similarly the lengths of successive undeleted runs (indeed all undeleted runs) are assumed to be independent. While we do not have a rigorous proof of these assumptions, they have been confirmed by extensive simulations.

Let *ϕ*_1 _and *ϕ*_2 _be the proportion of deletion events affecting homeologous chromosomes 1 and 2, respectively, so that *ϕ*_1 _+ *ϕ*_2 _= 1. Let *τ*(*r*) be the proportion of runs of single-copy genes with terms in both chromosomes. (*τ*(1) ≡ 0 and, initially, *τ*(*r*) = 0 for *r *= 2, 3, ⋯.) Note that in such a run, the term(s) at the extreme left were (was) deleted from chromosome *i *with probability *ϕ*_*i *_and the same for the terms at the extreme right.

The proportion of undeleted terms in runs of length *l *is *lρ*(*l*)/*E*_*ρ*_, where *E*_*ρ *_= ∑_*l*__>0 _*lρ*(*l*). As depicted in Figure 3, the probabilities $p_{A_{1}}$ and $p_{A_{2}}$ that a deletion event affects chromosomes 1 or 2, respectively, and falls within a run of undeleted terms of length *l *without deleting the terms at either end is, for *i *∈ {1, 2}

(2) $$\begin{array}{llll} p_{A_{i}}\; & =\;\phi_{i}\underset{l>2}{\sum }\frac{l\rho \left(l\right)}{E_{\rho }}\overset{l-1}{\underset{j-2}{\sum }}\frac{1}{l}\overset{l-j}{\underset{a=1}{\sum }}\gamma \left(a\right)\; & & \;\\ & =\;\frac{\phi_{i}}{E_{\rho }}\underset{l>2}{\sum }\rho \left(l\right)\overset{l-1}{\underset{j=2}{\sum }}\overset{l-j}{\underset{a=1}{\sum }}\gamma \left(a\right)\; & & \;\\ & =\;\frac{\phi_{i}}{E_{\rho }}\underset{l>2}{\sum }\rho \left(l\right)\overset{l-2}{\underset{a=1}{\sum }}\left(l-a-1\right)\gamma \left(a\right)\; & & \;\\ & \; & \end{array}$$

where *j *indexes the starting position of the deletion within the run, and *a *is the number of terms deleted in the event. We define the *contribution to mean run length *of *A *events to be

(3) $$\mu_{A}=\overset{2}{\underset{i=1}{\sum }}\frac{\phi_{i}}{E_{\rho }}\underset{l>2}{\sum }\rho \left(l\right)\overset{l-2}{\underset{a=1}{\sum }}\left(l-a-1\right)\gamma \left(a\right)a.$$

Events of type *A*_*i *_create runs of deleted terms with *r *= 1 from one chromosome only. Note that the last line of equation (2), and equation (3), involve the collection of terms, reducing the number of nested summations in order to speed up calculation. While these are not lengthy calculations to start with, we display the speed-up as a simple illustration of the important efficiencies implemented for more difficult cases to be treated below.

The probability $p_{B_{if}}$ that a deletion event on chromosome *i *touches only the run of deletions on chromosome *f *on the left of the run of undeleted terms is, for *i *∈ {1, 2} and *f *∈ {1, 2},

(4) $$p_{B_{if}}=\frac{\phi_{i}\phi_{f}}{E_{\rho }}\underset{l>1}{\sum }\rho \left(l\right)\overset{l-1}{\underset{a=1}{\sum }}\gamma \left(a\right).$$

We define the contribution to mean run length of *B *events to be

(5) $$\mu_{B}=\overset{2}{\underset{i=1}{\sum }}\overset{2}{\underset{f=1}{\sum }}\frac{\phi_{i}\phi_{f}}{E_{\rho }}\underset{l>1}{\sum }\rho \left(l\right)\overset{l-1}{\underset{a=1}{\sum }}\gamma \left(a\right)a.$$

Events of type *B*_*ii *_turn a deleted run with *r *events from one chromosome, into a run with *r *+ 1 events. Events of type *B*_*if*_, with *i *≠ *f*, turn a deleted run with *r *events, into a run with *r *+ 1 events.

The probability $p_{C_{ii}}$ that a deletion event, on either chromosome, does not touch the run of deletions on the left, does touch or overlap the run of deletions on the right entirely on the same chromosome (homeolog), but does not extend over the entire run of undeleted terms beyond that is, for *i *∈ {1, 2}:

(6) $$\begin{array}{llll} p_{C_{ii}}\; & =\;\frac{\phi_{i}^{2}\left(1-\tau \right)}{E_{\rho }}\underset{l>1}{\sum }\underset{k\geq 1}{\sum }\rho \left(l\right)\rho \left(k\right)\overset{l}{\underset{j=2}{\sum }}\overset{l-j+k}{\underset{a=l-j+1}{\sum }}\gamma \left(a\right)\; & & \;\\ & =\;\frac{\phi_{i}^{2}\left(1-\tau \right)}{E_{\rho }}\underset{l>1}{\sum }\underset{k\geq 1}{\sum }\rho \left(l\right)\rho \left(k\right)\; & & \;\\ & \times \;\left(\overset{\min\left[l-2,k-1\right]}{\underset{a=1}{\sum }}a\gamma \left(a\right)+\overset{\max\left[l-1,k\right]}{\underset{a=\min\left[l-1,k\right]}{\sum }}\min\left[l-1,k\right]\gamma \left(a\right)+\overset{l+k-2}{\underset{a=\max\left[l,k+1\right]}{\sum }}\left(l+k-a-1\right)\gamma \left(a\right)\right).\; & & \;\\ & \; & \end{array}$$

We define the contribution to mean run length of *C*_*ii *_events to be

(7) $$\mu_{C_{ii}}=\overset{2}{\underset{i=1}{\sum }}\frac{\phi_{i}^{2}\left(1-\tau \right)}{E_{\rho }}\underset{l>1}{\sum }\underset{k>1}{\sum }\rho \left(l\right)\rho \left(k\right)\overset{l}{\underset{j=2}{\sum }}\overset{l-j+k}{\underset{a=1-j+1}{\sum }}\gamma \left(a\right)a,$$

which can be calculated using an expansion such as that in (6). Events of type *C*_*ii *_turn a deleted run with *r *events from one chromosome, into a run with *r *+ 1 events.

The probability $p_{C_{if}}$ that a deletion event, on either chromosome, does not touch the run of deletions on the left but does touch the run of deletions on the right, partly or entirely on the other chromosome, is, for i ≠ *f *∈ {1, 2}:

(8) $$p_{C_{if}}=\frac{\phi_{i}\tau +\phi_{i}\phi_{f}\left(1-\tau \right)}{E_{\rho }}\underset{l>1}{\sum }\rho \left(l\right)\overset{l}{\underset{j=2}{\sum }}\overset{\infty }{\underset{a=l-j+1}{\sum }}\gamma \left(a\right).$$

We define the contribution to mean run length of *C*_*if *_events to be

(9) $$\mu_{C_{if}}=\overset{2}{\underset{i\neq f=1}{\sum }}\frac{\phi_{i}\tau +\phi_{i}\phi_{f}\left(1-\tau \right)}{E_{\rho }}\underset{l>1}{\sum }\rho \left(l\right)\overset{l}{\underset{j=2}{\sum }}\left(l-j+1\right)\overset{\infty }{\underset{a=l-j+1}{\sum }}\gamma \left(a\right).$$

Events of type *C*_*if*_, with *i *≠ *f*, turn a deleted run with *r *events, into a run with *r *+ 1 events. Note that (9) does not contains terms of form *aγ*(*a*) as do (3,5,7), since in this event deletion is blocked beyond the existing run of deletions; the probability weight is thus concentrated on deletions of lesser length.

The probability $p_{D_{iii}}$ that a deletion event completely overlaps the run of deletions on the right and touches or overlaps the run of deletions beyond that, all on the same chromosome, but does not extend over a further run of undeleted terms:

(10) $$\begin{array}{lll} p_{D_{iii}}\; & =\;\frac{\phi_{i}^{3}{\left(1-\tau \right)}^{2}}{E_{\rho }}\underset{l>1}{\sum }\underset{k\geq 1}{\sum }\underset{h\geq 1}{\sum }\rho \left(l\right)\rho \left(k\right)\rho \left(h\right)\overset{l}{\underset{j=2}{\sum }}\overset{l-j+k+h}{\underset{a=l-j+k+1}{\sum }}\gamma \left(a\right)\; & \text{(1)}\\ & =\;\frac{\phi_{i}^{3}{\left(1-\tau \right)}^{2}}{E_{\rho }}\underset{l>1}{\sum }\underset{k\geq 1}{\sum }\underset{h\geq 1}{\sum }\rho \left(l\right)\rho \left(k\right)\rho \left(h\right)\; & \text{(2)}\\ & \times \;\left(\overset{\min\left[l+k-2,h+k-1\right]}{\underset{a=k+1}{\sum }}\left(a-k\right)\gamma \left(a\right)+\overset{\max\left[l+k-1,k+h\right]}{\underset{a=\min\left[l+k-1,k+h\right]}{\sum }}\min\left[l-1,h\right]\gamma \left(a\right)+\overset{l+k+h-2}{\underset{a=\max\left[l+k,k+h+1\right]}{\sum }}\left(l+k+h-a-1\right)\gamma \left(a\right)\right)\; & \text{(3)}\\ & \; & \text{(4) } \end{array}$$

in which the reduction of the number of nested summations is key to the computability of the entire calculation.

We define the contribution to mean run length of *D*_*iii *_events to be

(11) $$\mu_{D_{iii}}=\frac{\phi_{i}^{3}{\left(1-\tau \right)}^{2}}{E_{\rho }}\underset{l>1}{\sum }\underset{k\geq 1}{\sum }\underset{h\geq 1}{\sum }\rho \left(l\right)\rho \left(k\right)\rho \left(h\right)\overset{l}{\underset{j=2}{\sum }}\overset{l-j+k+h}{\underset{a=1-j+k+1}{\sum }}\gamma \left(a\right)a,$$

which can be calculated using an expansion such as that in (10). Events of type *D*_*iii *_turn two deleted runs with *r *and *s *events, respectively, both from the same chromosome, into a run with *r *+ *s *+ 1 events.

The probability $p_{D_{iif}}$ that a deletion event completely overlaps the run of deletions on the right, on the same chromosome, and touches the run of deletions beyond that, partly or entirely on the other chromosome, is:

(12) $$p_{D_{iif}}=\frac{\phi_{i}^{2}\left(1-\tau \right)\tau +\phi_{i}^{2}\phi_{f}{\left(1-\tau \right)}^{2}}{E_{\rho }}\underset{l>1}{\sum }\underset{k\geq 1}{\sum }\rho \left(l\right)\rho \left(k\right)\overset{l}{\underset{j=2}{\sum }}\overset{\infty }{\underset{a=l-j+k+1}{\sum }}\gamma \left(a\right).$$

and the contribution to mean run length is

(13) $$\mu_{D_{iif}}=\frac{\phi_{i}^{2}\left(1-\tau \right)\tau +\phi_{i}^{2}\phi_{f}{\left(1-\tau \right)}^{2}}{E_{\rho }}\underset{l>1}{\sum }\underset{k\geq 1}{\sum }\rho \left(l\right)\rho \left(k\right)\overset{l}{\underset{j=2}{\sum }}\left(l-j+k+1\right)\overset{\infty }{\underset{a=l-j+k+1}{\sum }}\gamma \left(a\right).$$

Events of type *D*_*iif*_, with *i *≠ *f*, turn two deleted runs with *r *and *s *events, respectively, with the latter containing terms from both chromosomes, into a single run with *r *+ *s *+ 1 events.

The probability $p_{E_{iii}}$ that a deletion event touches the run of deletions on the left of the run of undeleted terms and touches or overlaps the run of deletions on the right, all on the same chromosome, but does not extend over the entire run of undeleted terms beyond that is:

(14) $$p_{E_{iii}}=\frac{\phi_{i}^{3}\left(1-\tau \right)}{E_{\rho }}\underset{l\geq 1}{\sum }\underset{k\geq 1}{\sum }\rho \left(l\right)\rho \left(k\right)\overset{l+k-1}{\underset{a=1}{\sum }}\gamma \left(a\right),$$

where

(15) $$\mu_{E_{iii}}=\frac{\phi_{i}^{3}\left(1-\tau \right)}{E_{\rho }}\underset{l\geq 1}{\sum }\underset{k\geq 1}{\sum }\rho \left(l\right)\rho \left(k\right)\overset{l+k-1}{\underset{a=l}{\sum }}\gamma \left(a\right)a.$$

The probability $p_{E_{iif}}$ that a deletion event touches the run of deletions on the left of the run of undeleted terms, both from the same chromosome, and touches the run of deletions on the right, partly or entirely on the other chromosome, is:

(16) $$p_{E_{iif}}=\frac{\phi_{i}^{2}\tau +\phi_{i}^{2}\phi_{f}\left(1-\tau \right)}{E_{\rho }}\underset{l\geq 1}{\sum }\rho \left(l\right)\overset{\infty }{\underset{a=l}{\sum }}\gamma \left(a\right)$$

and

(17) $$\mu_{E_{iif}}=\frac{\phi_{i}^{2}\tau +\phi_{i}^{2}\phi_{f}\left(1-\tau \right)}{E_{\rho }}\underset{l\geq 1}{\sum }\rho \left(l\right)l\overset{\infty }{\underset{a=l}{\sum }}\gamma \left(a\right).$$

The probability $p_{E_{iii}}$ that a deletion event touches the run of deletions on the left of the run of undeleted terms and touches or overlaps the run of deletions on the right, all on the same chromosome, but does not extend over the entire run of undeleted terms beyond that is:

(18) $$p_{E_{ifi}}=\frac{\phi_{i}^{2}\phi_{f}\left(1-\tau \right)}{E_{\rho }}\underset{l\geq 1}{\sum }\underset{k\geq 1}{\sum }\rho \left(l\right)\rho \left(k\right)\overset{l+k-1}{\underset{a=l}{\sum }}\gamma \left(a\right)$$

and

(19) $$\mu_{E_{ifi}}=\frac{\phi_{i}^{2}\phi_{f}\left(1-\tau \right)}{E_{\rho }}\underset{l\geq 1}{\sum }\underset{k\geq 1}{\sum }\rho \left(l\right)\rho \left(k\right)\overset{l+k=1}{\underset{a=l}{\sum }}\gamma \left(a\right)a$$

The probability $p_{E_{iff}}$ that a deletion event touches the run of deletions on the left of the run of undeleted terms and touches or overlaps the run of deletions on the right, all on the same chromosome, but does not extend over the entire run of undeleted terms beyond that is:

(20) $$p_{E_{iff}}=\frac{\phi_{i}\phi_{f}\tau +\phi_{i}\phi_{f}^{2}\left(1-\tau \right)}{E_{\rho }}\underset{l\geq 1}{\sum }\rho \left(l\right)\overset{\infty }{\underset{a=l}{\sum }}\gamma \left(a\right)$$

and

(21) $$\mu_{E_{iff}}=\frac{\phi_{i}\phi_{f}\tau +\phi_{i}\phi_{f}^{2}\left(1-\tau \right)}{E_{\rho }}\underset{l\geq 1}{\sum }\rho \left(l\right)l\overset{\infty }{\underset{a=l}{\sum }}\gamma \left(a\right)$$

Events of type *E*_*iii *_turn two deleted runs with *r *and *s *events, respectively, all from one chromosome, into a single run with *r *+ *s *+ 1 events. Events of type *E*_*iif*_, *E*_*ifi *_and E_*iff*_,, with *i *≠ *f*, turn two deleted runs with *r *and *s *events, respectively, into a single run with *r *+ *s *+ 1 events.

We reiterate here that the last lines of each of (2),(6) and (10) include the collection of terms, significantly cutting down on computing time when these formulae are implemented, especially in the case of (10).

In this initial model, we neglect the merger of three or more runs of deletions. There is no conceptual difficulty in including three or more mergers, but the proliferation of embedded summations would require excessive computation. Thus we should expect the model to be adequate until *θ *gets very small, when mergers of several runs at a time become common.

Let $p_{A}=p_{A_{1}}+p_{A_{2}}$, and similarly let each of *p*_*B*_, ⋯, *p*_*E *_be the sums of their respective subscripted terms (with all combinations of *i *and *f*). We define the change *δ*_*π*_(*r*) in the number of runs of deleted terms with *r *= 1, 2, ....

(22) $$\delta_{\pi }\left(1\right)=p_{A}-\left(p_{B}+p_{C}+2p_{D}+2p_{E}\right)\pi \left(1\right).$$

(23) $$\delta_{\pi }\left(2\right)=\left(p_{B}+p_{C}\right)\pi \left(1\right)-\left(p_{B}+p_{C}+2p_{D}+2p_{E}\right)\pi \left(2\right).$$

For *r *> 2,

(24) $$\delta_{\pi }\left(r\right)=\left(p_{B}+p_{C}\right)\pi \left(r-1\right)+\left(2p_{D}+2p_{E}\right)\overset{r-2}{\underset{s=1}{\sum }}\pi \left(s\right)\pi \left(r-s-1\right)-\left(p_{B}+p_{C}+2p_{D}+2p_{E}\right)\pi \left(r\right).$$

In an implementation on a finite interval of **ℤ**, the number of runs of deleted terms will change from some value *R *to *R'*, where

(25) $$R^{\prime }=R+\overset{\infty }{\underset{r=1}{\sum }}\delta_{\pi }\left(r\right).$$

The distribution of number of events per run will also change from *π *to *π'*, where

(26) $$\pi^{\prime }\left(r\right)=\frac{R\pi \left(r\right)+\delta_{\pi }\left(r\right)}{R^{\prime }},$$

and where the mean of the number of deleted genes per run increases from  to ${ū}^{\prime }$, so that

(27) $${ū}^{\prime }=\frac{Rū+\sum_{X=A,B,C.,D.,E.}\mu X}{R^{\prime }}.$$

The mean ${\bar{v}}^{\prime }$ of the new distribution *ρ' *of run lengths of undeleted terms satisfies

(28) $${\bar{v}}^{\prime }=\frac{R}{R^{\prime }}\left(ū+\bar{v}\right)-{ū}^{\prime }.$$

The new proportion *θ' *of undeleted terms is ${\bar{v}}^{\prime }∕\left({ū}^{\prime }+{\bar{v}}^{\prime }\right)$.

In the same interval of **ℤ**, we define the change *δ*_*τ*_(*r*) in the number of runs containing single copy genes in both chromosomes with *r *= 1, 2, ....

(29) $$\delta_{\tau }\left(1\right)=0.$$

(30) $$\delta_{\tau }\left(2\right)=\left(p_{B_{12}}+p_{B_{12}}+p_{C_{12}}+p_{C_{21}}\right)\pi \left(1\right)-\left(p_{B}+p_{C}+2p_{D}+2p_{E}\right)\pi \left(2\right)\tau \left(2\right).$$

For *r *> 2,

(31) $$\begin{array}{llll} \delta_{\tau }\left(r\right)\; & =\;\left(p_{B}+p_{C}\right)\pi \left(r-1\right)\tau \left(r-1\right)+\left(p_{B_{12}}+p_{B_{12}}+p_{C_{12}}+p_{C_{21}}\right)\pi \left(r-1\right)\left(1-r\left(r-1\right)\right)\; & & \;\\ & +\;\left(2p_{D}+2p_{E}\right)\overset{r-2}{\underset{s=1}{\sum }}\pi \left(s\right)\pi \left(r-s-1\right)\left(1-\left(\phi_{1}^{3}+\phi_{2}^{3}\right)\left[1-\tau \left(r-s-1\right)\right]\left[1-\tau \left(s\right)\right]\right)\; & & \;\\ & -\;\left(p_{B}+p_{C}+2p_{D}+2p_{E}\right)\tau \left(r\right)\pi \left(r\right).\; & & \;\\ & \; & \end{array}$$

In the implementation, the number of runs of deleted terms with genes on both chromosomes will change from *T*(*r*) to *T'*(*r*), where

(32) $$T^{\prime }\left(r\right)=T\left(r\right)+\delta_{\tau }\left(r\right).$$

The proportions of runs with deletion events from both chromosomes will also change from *τ *to *τ'*, where

(33) $$\tau^{\prime }\left(r\right)=\frac{T^{\prime }\left(r\right)}{R^{\prime }\pi^{\prime }\left(r\right)}.$$

We implement equations (1) to (33) as a recurrence with a step size parameter Λ to control the number of events using the same *p*_*A*_, *p*_*B*_, *p*_*C*_, *p*_*D*_, *p*_*E*_, *δ*_*π*_(·) and *δ*_*τ*_(·) between successive normalizations, and using Λ*δ*_*π*_(·) and Λ*δ*_*τ*_(·) instead of *δ*_*π*_(·) and *δ*_*τ*_(·) in (25)-(33). The choice of Λ determines the trade-off between computing speed and accuracy.

**Figure 4:**
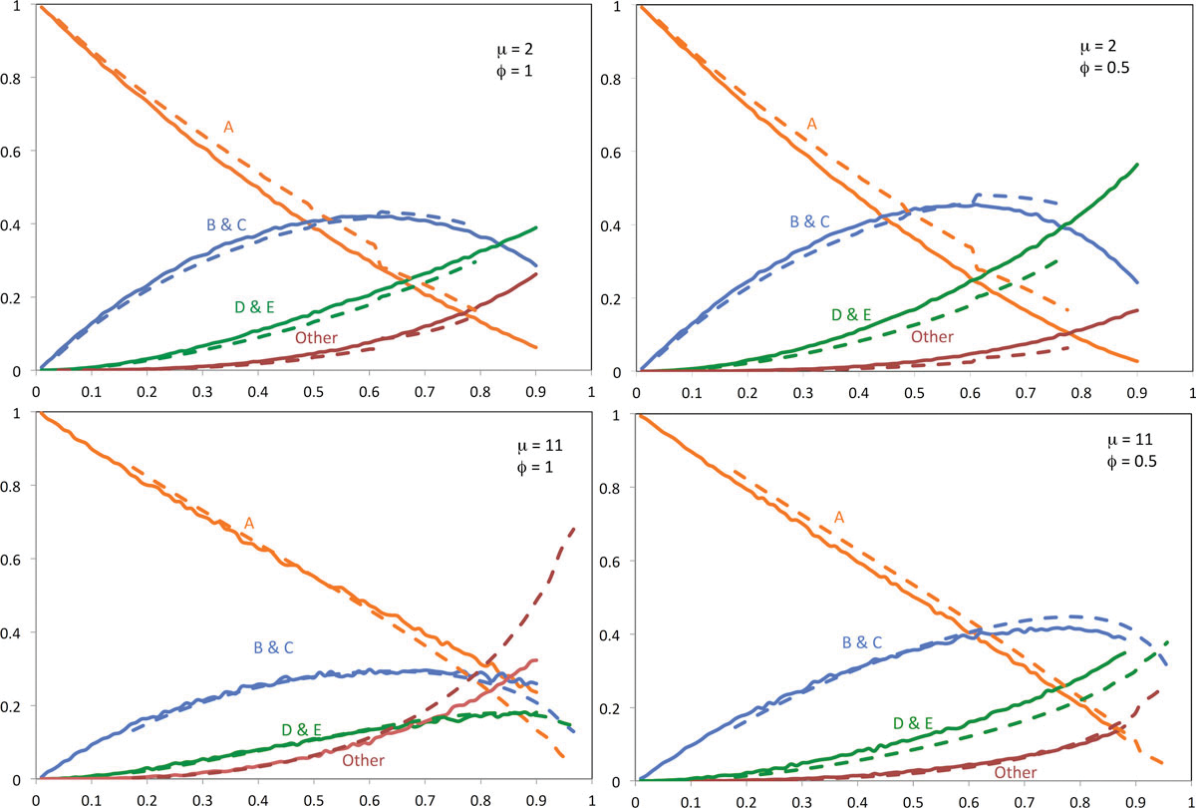
**Comparison of event frequencies in simulations and model.** Changes in rates of different event types as calculated by recurrence (dashed lines), compared with simulation results (solid lines). Horizontal axis: Proportion of duplicates deleted = 1 - *θ*. Vertical axis: proportion of event type.

Figure 4 shows the results of our current implementation of our deterministic recurrence for the cases *μ *= 2 and *μ *= 11, for unbiased fractionation (*ϕ *= 0.5) and for extremely biased fractionation (*ϕ *= 1). The results fit simulations of the stochastic model quite well and reveal a number of tendencies. One is that unbiased fractionation with small deletions leads to the fastest drop in events of type A as *θ *decreases.

Biased fractionation with large deletion sizes leads to slow initial growth in the proportions of events of types D and E and "other".

There are at least two reasons for the discrepancies between the simulations and the recurrences observed in Figure 4. At the outset, since we used a large step size Λ for the computationally costly recurrence, its trajectory lags behind the simulation, especially with respect to the slower decrease in *p*_*A *_and slower increase in *p*_*B *_+ *p*_*C*_. Later discrepancies are partially due to not accounting for the merger of three or more runs. These can be estimated and are summarized as "other " in the diagram, but the quantities involved are not fed back to the recurrence through (26).

Other possible sources of error might be due to the cutoffs in *x *used for calculations involving *γ*(*x*) and *ρ*(*x*). However, extensive testing of various cutoff values has indicated such errors to be negligible in our implementation.

## Conclusions

We have developed a model for the fractionation process based on deletion events excising a geometrically-distributed number of contiguous paralogs from either one of a pair of homeologous chromosomes. The existence of data prompting this model is due to a functional biological constraint against deleting both copies of a duplicate pair of genes.

The mathematical framework we propose should eventually serve for testing the geometric excision hypothesis against alternatives such as single gene-by-gene inactivations, although we have not developed this in this paper. In addition, further developments could treat the gene-by-gene inactivation model as the null hypothesis, and the geometric excision model, with mean greater than 1, as the alternative hypothesis.

Simulations of these models indicate the feasibility of estimating the mean *μ *of the deletion event process and the fractionation bias *ϕ *from observations of the length of runs of single-copy genes and the overall proportion of single-copy genes.

The main question we have explored is the exact derivation of *π*, the distribution of the number of deletion events contributing to a run of single-copy genes. The simulations are convenient in practice, since they depend on only the parameters *μ *and *ϕ *as they evolve over time, but they give little mathematical insight. Our most important advance is a deterministic recurrence for the *π*(*r*) as the proportion *θ *of undeleted genes decreases. This takes into account the appearance of new runs over time, the lengthening of existing runs, as well as the merger of two existing runs with the new deletions to form a single, longer one. This calculation fits the process as simulated rather well and seems promising for further development.

In order to validate our fractionation model empirically, we will have to expand it to incorporate the rearrangement events that are pervasive in genome evolution. Our previous work on this problem shows that the effect of rearrangement is to seriously bias the observable, credible instances of fractionation towards smaller runs of deleted genes [4,8]. Future work on this difficult problem will have either to rely on careful modeling of this ascertainment bias or else find a way to incorporate into the model deleted runs that have been interrupted by rearrangements.

## Competing interests

The authors declare that they have no competing interests.

## Authors' contributions

DS, CZ and BW formulated the problem, carried out the calculations and simulations, and wrote the paper. All authors read and approved the final manuscript.
